# Factors explaining individual differences in the oral perception of capsaicin, *l* ‐menthol, and aluminum ammonium sulfate

**DOI:** 10.1111/cts.13587

**Published:** 2023-07-25

**Authors:** Sulo Roukka, Sari Puputti, Heikki Aisala, Ulla Hoppu, Laila Seppä, Mari Sandell

**Affiliations:** ^1^ Department of Food and Nutrition University of Helsinki Helsinki Finland; ^2^ Functional Foods Forum University of Turku Turku Finland; ^3^ Present address: Valio Ltd. Helsinki Finland; ^4^ Present address: VTT Technical Research Centre of Finland Ltd. Espoo Finland

## Abstract

This research focused on the oral perception of naturally occurring chemical food compounds that are used in the pharma and food industries due to their pharmacological properties. They stimulate chemically sensitive receptors of the somatosensory system and are also chemesthetic compounds. Capsaicin is a naturally occurring alkaloid activating pungency perception. *l*‐Menthol is a cyclic monoterpene working also as a medical cooling agent. Aluminum ammonium sulfate is used as a dehydrating agent and additive known to activate astringency in oral cavity. The objective of the study was to identify factors explaining individual differences in the perception of oral chemesthesis measured as sensitivity to chemesthetic compounds and their recognition. The subjects (*N* = 205) evaluated quality‐specific prototypic compounds at five different concentration levels. Differences between gender were discovered in capsaicin sensitivity with men being less sensitive than women. Age was associated with the perception of capsaicin, *l*‐menthol, aluminum ammonium sulfate, and the combined oral chemesthetic sensitivity. Quality‐specific recognition ratings were also contributing to the sensitivity to chemesthetic compounds. A combined oral chemesthetic recognition score was created based on quality‐specific recognition ratings. Increasing age generally indicated weaker recognition skills. Better recognizers had a higher combined oral chemesthetic sensitivity score than poorer recognizers. These results provide new information about chemesthesis. The results suggest that age and gender are important factors in explaining individual differences in sensitivity to capsaicin, *l*‐menthol, and aluminum ammonium sulfate. In addition, recognition skills are associated with the sensitivity based on the quality‐specific recognition scores.

**Study Highlights**

**WHAT IS THE CURRENT KNOWLEDGE ON THE TOPIC?**

Chemesthetic compounds, such as capsaicin and *l*‐menthol, have pharmacological properties and are used in various products. Individual differences among human beings in the oral perception and sensitivity of capsaicin and *l*‐menthol exist. There is a gap in knowledge explaining individual differences in the oral perception of chemesthetic compounds.

**WHAT QUESTION DID THIS STUDY ADDRESS?**

Do personal factors explain individual differences in oral perception of naturally occurring chemical compounds that impact health?

**WHAT DOES THIS STUDY ADD TO OUR KNOWLEDGE?**

Insights for the role of factors explaining capsaicin, *l*‐menthol, and aluminum ammonium sulfate sensitivities and the recognition of these compounds in different concentrations among individuals. Age and gender are explaining chemesthetic compound elicited sensitivity and recognition.

**HOW MIGHT THIS CHANGE CLINICAL PHARMACOLOGY OR TRANSLATIONAL SCIENCE?**

Chemesthetic compounds are widely used in pharmacological products. They have an important role in human sensory experience and can affect health. Understanding individuality in chemesthetic perception and novel measuring methods will open new possibilities for pharmacological industry and beyond. Ethical product development should take into account individual differences in chemesthetic perception.

## INTRODUCTION

Chemesthesis is closely connected to our daily multisensory experiences impacting human health and well‐being.^1^ The word “chemesthesis” is defined as the sensitivity of mucosal surfaces to chemical compounds found in various pharmacological and hygiene products as well as in foods and beverages. A wide variety of chemesthetic sensations (such as astringent, burning, cooling, painful, pungent, and tickling), can be perceived in the human body when chemical compounds are directly activating somatosensory nerves.^1^, ^2^, ^3^, ^4^, ^5^, ^6^ The chemesthesis eliciting compounds are agonists to specific chemical receptors of the somatosensory system.^7^ In the oral cavity area, the transient receptor potential (TRP) family of cation channels is expressed in sensory nerve endings innervating the mucosa in epithelial cells.^8^ For instance, capsaicin (*e*)‐8‐methyl‐*N*‐vanillyl‐6‐nonenamide is a naturally occurring alkaloid and a pungent compound that selectively activates neurons through transient receptor potential cation channel subfamily V member 1.^9^, ^10^, ^11^ Another example of edible compounds having pharmacological and chemesthetic properties is *l*‐menthol that is a terpene and an agonist to the transient receptor potential cation channel subfamily M member 8 known as the primary cold sensor.^12^, ^13^ After the activation, the information of oral chemesthetic stimuli is conveyed through cranial nerves: trigeminal nerve, glossopharyngeal nerve, and vagus nerve into the somatosensory cortex where it is processed.^2^, ^4^, ^14^

The perception of chemesthesis varies based on the activating compounds. Capsaicin is primarily eliciting pungency and secondarily evoking bitterness in the oral cavity which can appear with a delay.^11^, ^15^, ^16^, ^17^ Cooling agent *l*‐menthol may activate peppermint odor, bitterness, and pungency.^18^ Too high concentration of *l*‐menthol in products may be perceived as an undesirable experience. Astringency is a complex and multifaceted sensation causing mouth and mucous membrane dryness and puckering.^5^ It is perceived with various chemicals, including polyphenolic compounds and metallic salts, such as aluminum ammonium sulfate [AlNH_4_(SO_4_)^2^].^5^, ^6^, ^19^ Wine, tea, berries, and vegetables are typical sources of polyphenolic compounds.^5^ Some astringent compounds can provoke a metallic sensation and evoke multiple transduction pathways, including taste, smell, and chemesthesis.^20^, ^21^ Chemosensory perception might be modified by the structure or other characteristics of the matrix, such as viscosity and sweetness.^22^

Individuals seem to vary in perception of chemesthetic stimuli. As an example, individual differences in capsaicin perception have been shown in several studies.^4^, ^23^, ^24^, ^25^ Our recent study showed that people could be classified into sensitivity groups based on their intensity ratings measured across different concentrations of chemesthetic compounds.^4^ In addition to oral cavity, studies on nasal chemesthesis suggest individual differences for capsaicin and *l*‐menthol.^5^, ^6^ Earlier research findings have discovered that age is not associated with capsaicin elicited pungency intensity.^26^, ^27^ A study on the intranasal trigeminal system (*N* = 16) suggested that older subjects (59–67 years) have a higher threshold and reduced sensitivity for *l*‐menthol compared to younger subjects (18–35 years).^22^ In another study (*N* = 29), men and women displayed similar threshold levels for *l*‐menthol when applied as a nasal chemesthetic stimulus.^28^ A research (*N* = 54) focusing on astringency perception showed that young adults (18–35 years) are more sensitive than the elderly (over 70 years) to tannic acid, based on the higher astringency threshold ratings.^19^

Chemesthetic compounds can promote health‐related benefits,^29^ including antibacterial activity,^30^, ^31^ anticancer effects,^13^, ^32^ cardiovascular protection,^33^, ^34^, ^35^ and anti‐obesity effects.^11^, ^36^, ^37^, ^38^, ^39^ In addition, their functional properties are having an important role in pain‐related medicine development because of the association with the stimulation of thermoreceptors and nociceptors.^40^, ^41^, ^42^ These beneficial impacts to physiology, pharmacology, nutrition, and metabolism are dependent on the compound's structure, sensory mechanisms, and desensitization sequences.^13^, ^32^, ^43^ To increase the ethical usage of chemesthetic compounds in pharmacological and food products, it is vital to understand the main factors explaining individual differences in the perception of chemesthesis. Surprisingly, there are not many studies focusing directly on the association between sensitivity to chemesthetic compounds and personal factors, such as gender and age.

Our recent chemesthesis study supports the existence of individual differences in sensitivity to chemesthetic stimuli.^4^ Moreover, a novel sensitivity score for chemesthesis was derived from compound‐based sensitivity groups. From the established data,^4^, ^44^, ^45^ we continued to investigate the same subjects and the background variables explaining individual differences in sensitivity. We hypothesized that personal factors,^45^ including intrinsic factors (gender and age), health‐related extrinsic factors (body mass index [BMI] and smoking status), and the capability to identify chemesthetic qualities, may potentially explain sensitivity to chemesthetic compounds.

## MATERIALS AND METHODS

### Subjects

A total of 205 Finnish‐speaking adult volunteers participated in the sensory study sessions.^4^, ^44^, ^45^ They were instructed not to consume foods, drinks other than water, smoke, or use mouth‐freshening products for 1 h before the sessions. Subject characteristics are shown in Table 1.^4^

**TABLE 1:** Subject characteristics, *N* = 205 (Puputti et al., 2019)^45^ and (Roukka et al., 2021).^4^

Variable		*N*	%	Data missing
Age^45^	41.7 ± 15.2	205	100	0
	19–34	88	42.9	
	35–49	59	28.8	
	50–79	58	28.3	
Gender^45^		205		0
	Female	164	80.0	
	Male	41	20.0	
BMI^45^	25.6 ± 5.6	198	96.6	7
	<25.0	111	56.1	
	25.0–29.9	51	25.8	
	≥30.0	36	18.2	
Smoking^45^		198		7
	Currently/former	51	25.8	
	Nonsmoker	147	74.2	
Capsaicin sensitivity^4^		199		6
	CSG1	56	28.1	
	CSG2	59	29.6	
	CSG3	84	42.2	
*l*‐Menthol sensitivity^4^		198		7
	MSG1	81	40.9	
	MSG2	96	48.5	
	MSG3	21	10.6	
AlNH_4_(SO_4_)^2^ sensitivity^4^		197		8
	ASG1	91	46.2	
	ASG2	62	31.5	
	ASG3	44	22.3	
Combined oral chemesthetic sensitivity^4^		196		9
	COCSG1	59	30.1	
	COCSG2	105	53.6	
	COCSG3	32	16.3	

*Note*: 1 = The least sensitive (hyposensitive). 2 = The semisensitive. 3 = The most sensitive (hypersensitive). CSG = Capsaicin sensitivity group (modified from Roukka et al., 2021^4^: P‐Abbreviations: A‐CSG, “Astringency chemesthetic sensitivity group”); ASG, AlNH_4_(SO_4_)^2^ sensitivity group (modified from Roukka et al. 2021; BMI, body mass index; C‐CSG, “Cooling chemesthetic sensitivity group”); COCSG, Combined oral chemesthetic sensitivity group; CSG, “Pungency chemesthetic sensitivity group”); MSG, *l*‐Menthol sensitivity group (modified from Roukka et al. 2021.^4^

### Ethical aspects

The study was institutional review board reviewed by the Southwest Finland Hospital District's Ethics Committee (145/1801/2014) and followed the European Union's General Data Protection Regulation guidelines. All the subjects signed a consent form that included detailed information about the structure of the study. Exclusion criteria included allergies, pregnancy, or lactating status.

### Chemical stimuli used in sensory evaluation

The chemesthetic qualities; pungency, cooling, and astringency were studied via one activating prototypic compound per each quality (Table 2).^4^ The quality‐specific series with six samples of differing concentrations were prepared. Each series included one water sample. The samples were prepared and stored under good laboratory practice in a cold room (+8°C) for less than 4 days before the evaluation, and then acclimatized at room temperature before serving.

**TABLE 2:** Chemesthesis sensory study samples.^4^

Chemesthetic quality	Prototypic compound	A	B	C	D	E
Pungent	Capsaicin^a^ (CAS: 404‐86‐4) ≥98.5% C_18_H_27_NO_3_	0.049 μM	0.088 μM	0.154 μM	0.275 μM	0.491 μM
Cooling	*l*‐Menthol^b^ (CAS: 2216‐51‐5) ≥99.7% C_10_H_20_O	0.013 mM	0.023 mM	0.040 mM	0.072 mM	0.128 mM
Astringent	Aluminum ammonium sulfate^c^ (CAS: 7784‐26‐1) ≥99.0% AlNH_4_(SO_4_)^2^ 12H_2_O	0.22 mM	0.39 mM	0.70 mM	1.24 mM	2.21 mM

*Note*: Dilutions: A–E.

^a^ Fluka Sigma‐Aldrich (St. Louis, MO, USA).

^b^ Symrise (Holzminden, Germany).

^c^ Produced by Sigma‐Aldrich (St. Louis, MO, USA).

### Sensory evaluation procedure

The study was conducted in the sensory laboratory (ISO‐8589 compliance) at the Functional Foods Forum, University of Turku, using Compusense five Plus 5.6 (Compusense) software. All the samples were served in glass beakers (30 mL) marked with three‐digit codes. The subjects were instructed to neutralize their mouths with water and a neutral unsalted cracker between the samples.

The subjects were untrained, but they were introduced to the chemesthetic qualities by tasting the second strongest sample of each sample set (D, in Table 2) before the data collection. The strongest sample (E, in Table 2) was offered, if the subject was not able to perceive the sensation of sample D. This pretesting was done to minimize the possible bias caused by the element of surprise in the actual evaluation and to introduce the subjects to the relevant terminology used in the evaluation.

The data collection started with the *l*‐menthol sample series. After a short break, the subjects evaluated the astringency sample series followed by the pungency series. The samples in each series (Table 2) were divided into two separately randomized lines that included dilutions from the lower (water, A, and B) and higher (C, D, and E) concentrations. This was done to prevent the element of desensitization which can occur especially when evaluating chemesthetic samples with higher concentrations of compounds.

The subjects tasted samples entirely (5 mL each) and evaluated the intensity of the stimuli using a line scale with labels from 0 to 10 (with supporting labels on the line scale 0 = “no sensation,” 2–3 = “very mild,” 4 = “quite mild,” 6 = “quite strong,” 8 = “very strong,” and 10 = “extremely strong”). Then the subjects were asked to select the best option to describe the perceived sensation from the given multiple‐choice questionnaire (pungent, cooling or even cold, astringent, metallic, and water). The samples were expelled after 5 s in the mouth. Due to the possible delay of the sensation, they were instructed to wait 10 s before rating and selecting the description.

### Background questionnaire

The subjects were asked to respond to a background questionnaire, including self‐reported variables with Webropol software (Webropol Inc). For gender, the options included male and female. Age was asked as a year of birth. For the BMI calculation, the subjects gave their weight in kilograms and their height in centimeters. Smoking was reported using scale daily, occasionally, have smoked before regularly, or I do not smoke.

### Sensitivity groups to chemesthetic compounds

The subjects were segmented earlier into three groups with hierarchical clustering based on their intensity ratings to chemesthetic compounds (Table 1).^4^ These compound‐specific groups were identified as “least sensitive (hyposensitive),” “semi‐sensitive,” and “most sensitive (hypersensitive).” In addition, combined sensitivity groups were formed based on their oral chemesthetic sensitivity score values from 1.00 “least sensitive” to 3.00 “most sensitive” that were created based on the compound‐specific sensitivity groups, including capsaicin (pungent), *l*‐menthol (cooling), and AlNH_4_(SO_4_)^2^ (astringent).^4^

### Oral chemesthetic quality recognition

The recognition data was dichotomized (1 = “correct recognition”; 0 = “not recognized”) after the subjects had named each sample. “Correct” recognition (e.g., in a series of *l*‐menthol samples) meant that the subjects selected the cooling option from the given list.

For each quality of five concentrations, a quality‐specific recognition value with an integer range of 0–5 was calculated. The average of the total correct number of chemesthetic quality was then combined and divided by the number of qualities involved in order to create a general model for each subject to describe their capability to recognize chemesthetic qualities. This combined value was named as the oral chemesthetic recognition score having a maximum score of 5.00 (all qualities and concentrations correctly identified). This score was individual for each subject who were then classified into chemesthetic recognition groups based on their recognition score as “better recognizers (BRs; 4.00–5.00),” “poorer recognizers (PRs; 0–2.00),” and “average recognizers (ARs; 2.33–3.67).”

### Data analysis

The association between (1) chemesthetic sensitivity and recognition, and (2) background variables gender, age, BMI, and smoking status (Table 1) were studied with chi‐square, Kruskal–Wallis, Mann–Whitney *U*, and Spearman's rank correlation tests. All the possible two‐way interactions with background variables were analyzed using both parametric and nonparametric approaches. However, only nonstatistically significant results were discovered from two‐way interactions, and, thus, the analysis focused on the main effects. Water samples were excluded from the analyses.

Multinomial logistic regression models were created by setting age, gender, BMI, smoking status, and chemesthetic quality‐specific recognition rate as predicting factors explaining chemesthetic sensitivity. The odds ratio (OR) indicates a relative risk ratio between the comparison group and the reference group of the predictor variable to fall in the comparison group rather than in the reference group of the dependent variable when adjusted with other factors also in the regression models. Statistical significances (nonsignificant, *p* ≤ 0.05 = *, *p* ≤ 0.01 = **, and *p* ≤ 0.001 = ***) are marked after the OR values. Continuous variables: age and BMI, were also studied separately with multinomial logistic regression model as grouped variables (age: “youngest 19–34 years,” “middle‐aged 35–49 years,” and “oldest 50–79 years”; BMI: “lean individuals <25.0,” “overweight individuals 25.0–29.9,” and “obese individuals ≥30.0).”

SPSS Statistics 27.0 (IBM Corporation) was used for the analyses with significance level of *p* ≤ 0.05. Some of the subjects did not complete every section of the study. Thus, the number of subjects (*N*‐values) and any missing data are dealt with in each analysis rather than entirely excluding the subjects from the study.

## RESULTS

### Subject characteristics within sensitivity groups

The distribution of capsaicin sensitivity differed by gender ($\chi_{\left[2\right]}^{2}$ = 7.8, *p* ≤ 0.05) suggesting that there were more men in the hyposensitive group than women and more women in the hypersensitive group than men. In addition, different distributions between *l*‐menthol sensitivity in age (*H*_[2]_ = 6.0, *p* ≤ 0.05) were detected suggesting hypersensitive (MSG3: 45.2 ± 11.8) individuals were generally older than semi‐sensitive (MSG2: 39.5 ± 14.5) subjects. Differences were also discovered between combined oral chemesthetic sensitivity and age (*H*_[2]_ = 7.3, *p* ≤ 0.05) suggesting hypersensitive subjects (COCSG3: 46.0 ± 14.6) were generally older than semi‐sensitive (COCSG3: 38.9 ± 13.1) subjects.

### Distributions of the oral chemesthetic recognition

**FIGURE 1:**
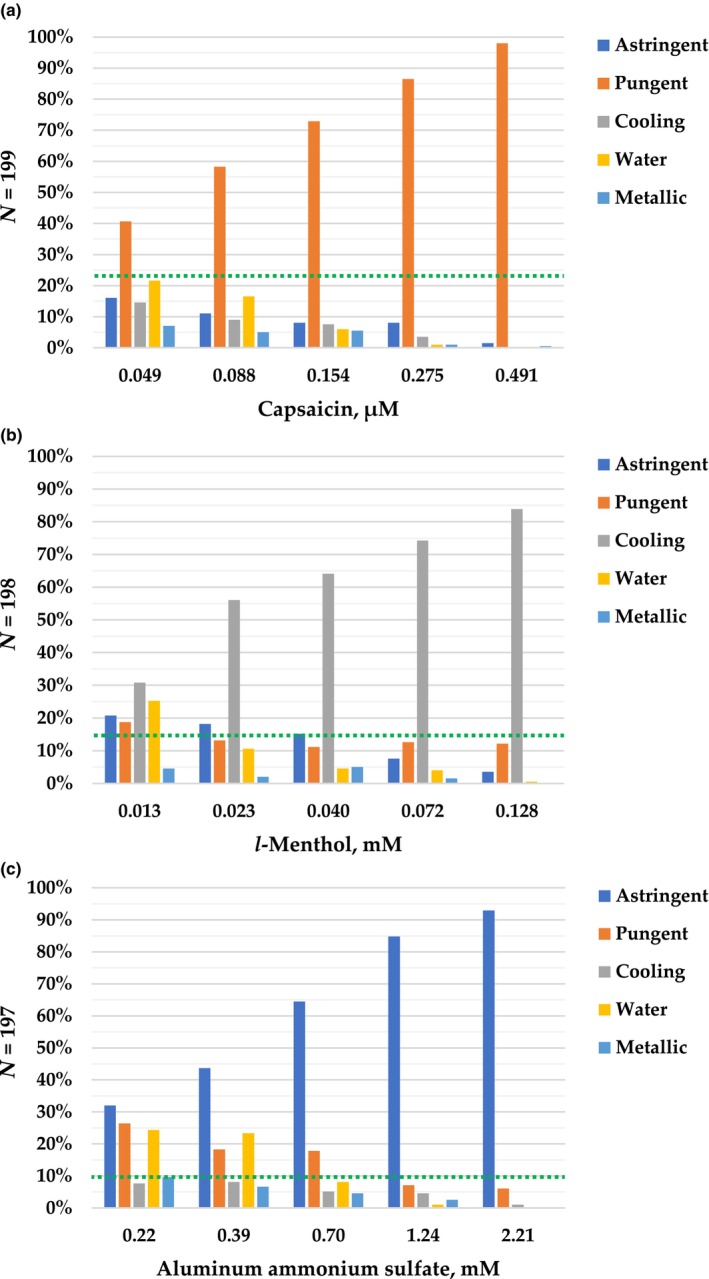
Oral chemesthetic quality recognition results for (a) capsaicin, (b) *l*‐menthol, and (c) aluminum ammonium sulfate solutions. The subjects were able to choose only one quality per sample (astringent, pungent, cooling, metallic, or water). The green dotted line informs the level of subjects who selected (a) only pungent from capsaicin samples (23.1%), (b) only cooling from *l*‐menthol samples (14.6%), and (c) only astringent from aluminum ammonium sulfate samples (9.6%).

The distributions of recognition responses for different prototypic chemesthetic compounds are presented in Figure 1: (a) capsaicin, (b) *l*‐menthol, and (c) AlNH_4_(SO_4_)^2^. Based on these graphs, the recognition rate increased in pungency, cooling, and astringency with higher concentrations.

The majority of the subjects recognized the four strongest capsaicin samples (B, C, D, and E) as pungent (Figure 1a). In the mildest concentration of capsaicin (A), the other three most frequently selected responses were water, astringent, and cooling. In the strongest and second strongest samples, the most frequently selected response other than pungent was astringent. Almost every subject (98%) selected the strongest capsaicin sample as pungent. Among all the chemesthetic qualities, pungency had the highest level of subjects (23.1%) who selected the correct (in this case, pungent) option for each concentration.

In the case of *l*‐menthol (Figure 1b), the majority recognized the four strongest samples (B, C, D, and E) as cooling. Only 14.6% of the subjects selected cooling for all samples (A–E). Depending on the subject the mildest *l*‐menthol was cooling, water, pungent, or astringent. The second mildest and mid‐level samples were astringent instead of cool for more than 15% of the subjects. Although the strongest and the second strongest samples were cooling for the majority of subjects, they were pungent for more than 10% of the subjects. The strongest *l*‐menthol sample was selected as cooling by the highest percentage (83.8%) of subjects.

Most of the subjects selected the three strongest AlNH_4_(SO_4_)^2^ samples (C, D, and E) as astringent (Figure 1c). The mildest sample (A) was selected to be astringent, pungent, metallic, or water. Only 9.6% of the subjects selected each concentration to be astringent. In the second mildest sample (B), close to 20% of subjects selected either pungent or water option. Some subjects perceived the strongest sample of AlNH_4_(SO_4_)^2^ as pungent and cooling rather than astringent. Yet, the strongest sample was astringent for the most (92.9%) subjects.

Age had negative correlations with cooling recognition (*ρ* = −0.3, *p* ≤ 0.001) and with astringency recognition (*ρ* = −0.2, *p* ≤ 0.05). This weak negative association indicated that older subjects may have weaker recognition skills in the cooling of *l*‐menthol and astringency of AlNH_4_(SO_4_)^2^.

### Oral chemesthetic recognition score

**FIGURE 2:**
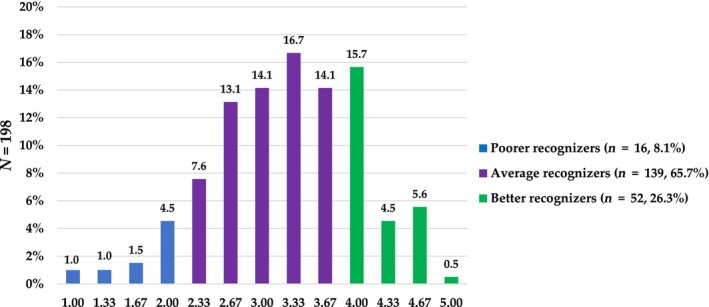
The distribution of oral chemesthetic recognition score (0–5.00) created from astringent, pungent, and cooling recognition results (*N* = 198). Getting 0 in this score is theoretically possible, however, none of the subjects got 0 correct.

The oral chemesthetic recognition score (mean ± standard deviation: 3.3 ± 0.8; *N* = 198) indicating the combined capability to recognize all the studied chemesthetic qualities (pungency, cooling, and astringency) was created, as seen in Figure 2. The highest number of subjects (65.8%) were ARs, whereas the minority (7.7%) were PRs, and the rest (26.5%) were BRs. When all correct answers were summed up from all the chemesthetic qualities (15 samples; pungency, cooling, and astringency; 5 concentrations each), the average of correct answers was 10, the minimum three, and the maximum 15.

Age was associated with the combined oral chemesthetic recognition score (*ρ* = −0.2, *p* ≤ 0.001). This weak negative correlation may indicate that older subjects have weaker general recognition skills for chemesthesis.

Oral chemesthetic recognition groups (PR, AR, and BR) had different oral chemesthetic sensitivity scores. BRs (2.1 ± 0.6) differed from both PRs (1.6 ± 0.7) and ARs (1.8 ± 0.6; *H*_[196]_ = 9.5, *p* ≤ 0.01).

### Factors linked to compound‐specific sensitivity

In the case of capsaicin sensitivity, a multinominal logistic regression model (Table 3) revealed that men were 3.07* times more likely than women to belong to CSG1 than CSG3. In addition, the higher pungent recognition rate indicated more sensitivity. As an example, people with higher pungent recognition rating would be 12.50** times (OR = 1/0.08**) more likely to be in CSG3 than CSG1. People with higher recognition scores would be 11.11** times more likely to belong to CSG3 than CSG2. When aging, each additional year increases the odds of belonging to CSG1 than CSG2 by 3% (OR = 1.03*). If age was handled as grouped variable, oldest subjects (50–79 years) were 2.64* times more likely to belong to CSG1 than CSG2 when comparing to the youngest group (19–34 years).

**TABLE 3:** Results of a multinomial logistic regression predicting capsaicin sensitivity *N* = 199 with subject characteristics (gender, age, BMI, and smoking status) and pungent recognition.

−2 Log likelihood 394.24, *χ* ^2^ (10) = 28.71, *p* = 0.001			
Nagelkerke Pseudo *R* ^2^: 0.161			
Goodness of fit: ns			
Capsaicin sensitivity (*n* = 199)	CSG1, ref CSG3	CSG2, ref CSG3 OR	CSG1, ref CSG2
	OR (95% CL)	(95% CL)	OR (95% CL)
Male^a^	3.07* (1.19–7.92)	2.35 ns (0.90–6.19)	1.31 ns (0.55–3.13)
Age (19–79 years)	1.01 ns (0.99–1.04)	0.98 ns (0.96–1.01)	1.03* (1.00–1.06)
BMI (15.9–47.9)	0.99 ns (0.93–1.08)	1.06 ns (0.99–1.13)	0.95 ns (0.88–1.02)
Smokers and former smokers^b^	1.32 ns (0.54–3.23)	1.57 ns (0.67–3.69)	1.31 ns (0.55–3.13)
Pungent recognition	0.08** (0.02–0.46)	0.09** (0.02–0.48)	0.97 ns (0.17–5.43)
Correctly predicted (%)	**48.5**		

*Note*: Odds ratios (95%, CL = “confidence level”) for all pairs of sensitivity groups and model fit statistics are displayed. Variables having a significant main effect in the model are bolded. Capsaicin sensitivity groups: CSG1 = least sensitive, CSG2 = semisensitive, and CSG3 = most sensitive. The largest (CSG3) and the second largest (CSG2) sensitivity groups were selected as references for the model. Significant results are bolded: *p ≤ 0.05, **p ≤ 0.01, and nonsignificant.

Abbreviations: BMI, body mass index; ns, nonsignificant; OR, odds ratio.

^a^ Reference category: Female.

^b^ Reference category: Nonsmokers.

The model for *l*‐menthol sensitivity (Table 4) showed that subjects with higher cooling recognition ratings were more sensitive. Those with higher cooling recognition ratings were 25.39** times more likely to belong to MSG3 than MSG1, and 9.09** times more likely to be in MSG2 than MSG1. Increasing age was also explaining *l*‐menthol sensitivity with an OR of 1.04*. This means the odds of belonging to MSG3 than MSG2 are increased by 4% for each additional year. If age was handled as a grouped variable, then middle‐aged subjects (35–49 years) were 7.38** times more likely to be MSG3 than MSG2, and 7.12* times MSG3 than MSG1 when compared to younger subjects (19–34 years). In addition, older subjects (50–79 years) were 5.35* times more likely to belong to MSG3 than MSG2 when compared to younger subjects.

**TABLE 4:** Results of multinomial logistic regression predicting *l*‐menthol sensitivity *N* = 198 with subject characteristics (gender, age, BMI, and smoking status) and cooling recognition.

−2 Log likelihood 341.84, *χ* ^2^ (10) = 25.26, *p* = 0.005			
Nagelkerke Pseudo *R* ^*2*^: 0.143			
Goodness of fit: ns			
*l*‐Menthol sensitivity (*n* = 198)	MSG1, ref MSG2	MSG3, ref MSG2	MSG3, ref MSG1
	OR (95% CL)	OR (95% CL)	OR (95% CL)
Male^a^	1.16 ns (0.54–2.51)	0.19 ns (0.02–1.59)	0.17 ns (0.02–1.39)
Age (19–79 years)	1.01 ns (0.99–1.03)	1.04* (1.00–1.08)	1.03 ns (0.99–1.07)
BMI (15.9–47.9)	1.00 ns (0.94–1.06)	0.95 ns (0.85–1.05)	0.95 ns (0.85–1.06)
Smokers and former smokers^b^	0.92 ns (0.43–1.97)	1.93 ns (0.60–6.21)	0.17 ns (0.02–1.39)
Cooling recognition	0.11** (0.03–0.44)	2.68 ns (0.26–27.79)	25.39** (2.22–290.72)
Correctly predicted (%)	**55.4**		

*Note*: Odds ratios (95%, CL = “confidence level”) for all pairs of sensitivity groups and model fit statistics are displayed. Variables having a significant main effect in the model are bolded. *l*‐Menthol sensitivity groups: MSG1 = least sensitive, MSG2 = semisensitive, and MSG3 = most sensitive. The largest (MSG2) and the second largest (MSG1) sensitivity groups were selected as references for the model. Significant results are bolded: * *p* ≤ 0.05, ** *p* ≤ 0.01, and ns = nonsignificant.

Abbreviations: BMI, body mass index; ns, nonsignificant.

^a^ Reference category: Female.

^b^ Reference category: Nonsmokers.

The model for AlNH_4_(SO_4_)^2^ (Table 5) showed that the astringency recognition rating was an explaining factor for sensitivity. A higher recognition rating predicted more sensitivity to AlNH_4_(SO_4_)^2^. People with higher astringent recognition ratings were 12.92** times more likely to belong to ASG2 than ASG1. In addition, they were 9.90* times more likely to belong to ASG3 than ASG1. Aging was also explaining AlNH_4_(SO_4_)^2^ sensitivity. The odds of people belonging to ASG3 than ASG2 are increased by 4% per added year (OR = 1.04*).

**TABLE 5:** Results of a multinomial logistic regression predicting AlNH_4_(SO_4_)^2^ sensitivity *N* = 197 with subject characteristics (gender, age, BMI, and smoking status) and astringent recognition.

−2 Log likelihood 232.16, *χ* ^2^ (14) = 23.69, *p* = 0.050			
Nagelkerke Pseudo *R* ^2^: 0.131			
Goodness of fit: ns			
AlNH_4_(SO_4_)^2^ sensitivity	ASG2, ref ASG1	ASG3, ref ASG1	ASG3, ref ASG2
(*N* = 197)	OR (95% CL)	OR (95% CL)	OR (95% CL)
Male^a^	0.54 ns (0.22–1.32)	0.55 ns (0.20–1.52)	1.01 ns (0.32–3.20)
Age (19–79 years)	0.98 ns (0.96–1.00)	1.02 ns (0.99–1.04)	1.04* (1.01–1.07)
BMI (15.9–47.9)	1.00 ns (0.94–1.07)	0.96 ns (0.88–1.04)	0.96 ns (0.88–1.04)
Smokers and former smokers^b^	1.02 ns (0.45–2.30)	0.81 ns (0.31–2.13)	0.79 ns (0.28–2.22)
Astringent recognition	10.66** (1.89–60.16)	9.40* (1.45–60.99)	0.88 ns (0.12–6.71)
Correctly predicted (%)	**50.0**		

*Note*: Odds ratios (95%, CL = “confidence level”) for all pairs of sensitivity groups and model fit statistics are displayed. Variables having a significant main effect in the model are bolded. AlNH_4_(SO_4_)^2^ sensitivity groups: ASG1 = least sensitive, ASG2 = semisensitive, and ASG3 = most sensitive. The largest (ASG1) and the second largest (ASG2) sensitivity groups were selected as references for the model. Significant results are bolded: * *p* ≤ 0.05, ** *p* ≤ 0.01, and nonsignificant.

Abbreviations: BMI, body mass index; ns, nonsignificant.

^a^ Reference category: Female.

^b^ Reference category: Nonsmokers.

### Predicting factors of combined oral chemesthetic sensitivity score

Gender differed significantly in combined oral chemesthetic sensitivity score (*U*_[196]_ = 2221.0, *p* ≤ 0.05). By average, women had 0.3 units higher score value (1.9 ± 0.6; *N* = 156) than men (1.6 ± 0.5; *N* = 38).

## DISCUSSION

This study focused on the sensory perception of chemical compounds that are widely used in the pharma and food industries because of their pharmacological benefits. According to these results, the main factors explaining sensitivity to chemesthetic compounds included in the study are gender, age, and chemesthetic quality‐specific recognition scores. This supports the hypothesis that similar factors explaining taste sensitivity^45^ can also explain chemesthetic sensitivity.^4^ Association between gender and combined oral chemesthetic sensitivity score indicated that women are more sensitive than men. Age was treated as a continues variable (Tables 3, 4, 5) which indicate yearly predictability. Every increase in the age range functions as an odds multiplier highlighting that age is a clinically important factor. In addition, the graphic recognition illustrations (Figure 1) showed that confusion of the samples was detected in each quality and sample concentration. The combined oral chemesthetic recognition score was formed. We were able to classify individuals into PR, AR, and BR based on their combined oral chemesthetic recognition score ratings. Overall, BRs were more sensitive than ARs and PRs based on their combined oral chemesthetic sensitivity scores. However, increasing age was found to cause weaker recognition skills.

In the case of capsaicin, women were more sensitive than men (Table 3). In a previous study, sensitivity in capsaicin elicited pungency perception has shown to be linked to responsiveness to PROP (6‐*n*‐propylthiouracil), and gender being the primary factor followed by PROP‐status contributing to sensitivity of pungency. Moreover, clinical research in South Korea on patients with chronic cough showed that elderly women were more sensitive to capsaicin among adults.^46^ In contrast to earlier studies,^26^, ^27^ we found that increasing age as a continuous variable explained capsaicin sensitivity (Table 3) suggesting sensitivity decreases when getting older. Additionally, when age was treated as a grouped variable, the oldest subjects were more likely to be less sensitive than younger subjects. A relationship between recognition of capsaicin as pungent and capsaicin sensitivity was found. Capsaicin samples were identified as pungent more often than other chemesthetic qualities, however, there were confusions detected especially in lower concentrations. These gender and age‐linked associations should be accounted for in capsaicin‐linked oral area treatments, such as topical capsaicin rinse therapy used for patients with burning mouth syndrome when choosing a suitable concentration level of capsaicin rinse.^47^

The results concerning *l*‐menthol showed that sensitivity groups differed with age. Older subjects are more sensitive to *l*‐menthol (Table 4). Increasing age also indicated poorer cooling recognition skills. Some subjects detected *l*‐menthol as pungent rather than cooling in higher concentrations (Figure 1b). *l*‐Menthol is known to be associated with pungency sensation in higher concentrations, which could indicate that more sensitive people might identify *l*‐menthol as pungent instead of cooling.^18^ Furthermore, a relationship between the recognition of *l‐*menthol as cooling and *l‐*menthol sensitivity was discovered. Individual differences in *l‐*menthol sensitivity in oral region have been discovered before with a relatively smaller subject number (*N* = 15).^23^ To our knowledge these personal factors explaining individual differences in oral *l‐*menthol sensitivity have not been reported in a larger human‐related study set. The customized product development in the pharma industry producing oral products and other industries should take into account these findings of individuality by creating alternative *l*‐menthol containing products (e.g., toothpaste and drugs)^13^, ^31^ with a lower concentration level, if wanting to sustain the products cooling‐induced sensation capabilities as humanely as possible.

Sensitivity to AlNH_4_(SO_4_)^2^ was explained with age as a continuous variable. Increasing age predicted that people are more sensitive to AlNH_4_(SO_4_)^2^ (Table 5). Individual differences in astringency sensitivity have been studied with different substances. For example, a tannic acid elicited astringency sensitivity study has discovered differences between older and younger subjects suggesting the former display a higher astringency threshold.^19^ The research also hypothesized that the salivary properties may influence astringency sensitivity as a function of age. Another study reported that gender is not a primary determinant of intensity of astringency.^48^ In our research, a relationship between AlNH_4_(SO_4_)^2^ sensitivity and recognition of AlNH_4_(SO_4_)^2^ as astringent was found (Table 5). Higher recognition score indicated higher sensitivity to AlNH_4_(SO_4_)^2^. Some subjects reported AlNH_4_(SO_4_)^2^ to be metallic rather than astringent. This might be because AlNH_4_(SO_4_)^2^ has a link to the metallic sensation via aluminum.^6^

Studies on oral chemesthesis are challenging to compare due to the multiple different compounds and methods used to evaluate the intensity or recognition. As an example, capsaicin elicited pungency perception can also be studied from mucous membranes with different techniques such as swabs,^15^ stripes,^17^ or liquid.^4^ In addition, some studies that measure astringency intensity have used tannic acid instead of AlNH_4_(SO_4_)^2^ or used a different set of concentrations.^19^ The intensity scales may also differ depending on the study. Therefore, standardized methods to measure the responses to chemesthetic perception would bring more convergent results and allow comparisons. This study highlights that careful planning is needed when conducting a sensory study, because these chemesthetic compounds are associated with pain perception, especially in high concentrations.^4^

Chemesthetic stimuli can rise and develop during time. Capsaicin and *l*‐menthol are capable of self‐desensitization and they can have cross‐desensitization effects.^23^ Thus, when serving multiple samples with different concentrations and cross‐desensitizing compounds, the resting period between the sensory stimuli must be long enough to prevent possible desensitization effects. These possible bias effects and other previously reported limitations considering the self‐reported factors are respectfully noted in this research.^4^, ^44^, ^45^ Here, the study design was planned and structured to prevent fatigue and keep subjects alert during the sensory study.

The generalized combined chemesthetic sensitivity and recognition scores can be extended with other chemesthetic qualities and substances.^20^ Although extension of the generalized models could increase its validity, having multiple different samples is challenging for subjects, and, in addition, the need for resting periods to neutralize senses is required.^4^ Our research, however, included several samples at different concentration levels with reagents that are proven chemesthetic quality‐specific agonists.^4^ The concentrations were carefully pretested for this research but can be further optimized based on our findings.

We suggest that the background factors should be studied with other chemesthetic compounds with improved sensory testing protocol. Our study provides relevant information about sensitivity to chemesthetic compounds and will increase the fundamental knowledge of factors explaining individual differences in oral chemesthetic sensitivity and recognition. The information and created novel score models could be utilized in various applications including pharmacology (e.g., drug development and side effect studies), medicine (e.g., pain, obesity, cardio, and cancer studies) as well as food (e.g., spices) and nonfood product development (e.g., hygiene products), and sustainable diet planning.

## AUTHOR CONTRIBUTIONS

S.R., S.P., H.A., U.H., L.S., and M.S. wrote the manuscript. S.P., H.A., U.H., and M.S. designed the research. S.P., H.A., and M.S. performed the research. S.R. analyzed the data. S.R., S.P., H.A., and M.S. contributed new analytical tools.

## FUNDING INFORMATION

This research was funded by the Academy of Finland (MAS 309408), the University of Helsinki (Faculty of Agriculture and Forestry), and the University of Turku (Faculty of Medicine).

## CONFLICT OF INTEREST STATEMENT

The authors declare no competing interests for this work.

## Data Availability

Data is available on reasonable request directed at the corresponding author.
